# Effectiveness of various communication strategies for improving childhood pneumonia case management: a community based behavioral open labeled trial in rural Lucknow, Uttar Pradesh, India

**DOI:** 10.1186/s12889-019-8050-0

**Published:** 2019-12-23

**Authors:** Shally Awasthi, Divas Kumar, Neha Mishra, Monika Agarwal, Chandra Mani Pandey

**Affiliations:** 1 Department of Pediatrics, King George’s Medical University, Lucknow, Uttar Pradesh India; 2 Department of Social and Preventive Medicine, King George’s Medical University, Lucknow, Uttar Pradesh India; 3 Department of Biostatistics and Health Informatics, Sanjay Gandhi Post Graduate Institute of Medical Sciences, Lucknow, Uttar Pradesh India

**Keywords:** Community acquired pneumonia, Behavior change, Qualified health care seeking, India, Trial

## Abstract

**Background:**

Community acquired pneumonia is responsible for 16% of under 5 mortality in India, probably due to delayed recognition and qualified care seeking. Therefore these deaths could possibly be averted by creating community awareness and promoting care seeking from qualified physicians in the government system. The objective of study was to assess the effectiveness of facility-based and village-based behavior change communication interventions delivered to community using validated information, education and communication materials, along with infrastructural strengthening of health facilities, for change in care seeking from government system for community acquired pneumonia in rural Lucknow, India.

**Method:**

Community based open labeled behavioral trial in 2 by 2 factorial design was conducted in eight rural blocks of Lucknow, northern India. Trained community health workers conducted Pneumonia Awareness Sessions once a month for the care givers of children using validated information, education and communication materials either at the villages or at government health facilities. Prior infrastructural strengthening of public health facilities was done to provide optimal care to cases. Pre packed pneumonia drug kits were provided which had amoxicillin, paracetamol and an instruction card on their use as well as pictorial representation of danger signs of pneumonia.

**Results:**

Study lasted from October 2015 to September 2018. Adherence to conduct of facility-based intervention was 93.0% (279/300) and to village-based intervention was 73.4% (7638/10410). In village-based intervention there was 79.3% (*p* < 0.0001) increase from a baseline of 3.3% (14/420) and facility-based intervention 68.9% (*p* = 0.02) increase from a baseline of 5.35% (21/392) in cases of possible pneumonia treated at government health facilities.

**Conclusion:**

Conduct of structured pneumonia awareness session using validated information, education and communication material at village level with infrastructural strengthening resulted in improved qualified care seeking from government facilities for community acquired pneumonia.

**Trial registration:**

AEARCTR-0003137, retrospectively registered on 10/July/2018.

## Background

Pneumonia is the leading cause of morbidity and mortality in children less than 5 years of age worldwide, with largest burden in developing countries like India. In 2016, 880,000 deaths of children under 5 years of age was reported due to pneumonia globally [1]. In India 0.18 million deaths in children in this age group was reported due to pneumonia in the year 2015 [2].

World Health Organization (WHO) defines community acquired pneumonia (CAP) as presence of fast breathing and/or chest in drawing. Fast breathing for children between 2 to 11 of age is 50 or more breaths per minute (bpm) and for 12–59 months of age is 40 or more bpm. WHO has classified children having fast breathing with or without chest in-drawing as “pneumonia” and as severe pneumonia when there were one or more general danger signs, such as inability to drink, persistent vomiting, convulsions, lethargy and unconsciousness [3].

After extensive formative research in Uttar Pradesh and Bihar states in northern India by the Principal Investigator (SA), it was found that (a) delay in recognition of symptoms, (b) delay in timely and qualified care seeking and (c) distrust of community in public health systems were probably the main reasons for increased mortality and morbidity due to childhood pneumonia [4]. Thereafter validated text, audio and video messages were developed to promote early symptom recognition and understand the importance of early and qualified care seeking for CAP [5].

Community care providers of children were then approached, either through facility-based or village-based behavior change intervention. The objective was to assess the effectiveness of delivering facility-based or village-based behavior change communication interventions using validated information, education and communication (IEC) materials, along with infrastructural strengthening of health facilities, for change in care seeking from government system for CAP in rural Lucknow.

Lucknow has a crude birth rate of 18.8 [6], infant mortality rate of 45 per 1000 live births [6] as compared to 41 per 1000 live births in India [7]. Under 5 mortality rate here is 60 per 1000 live births [6] as compared to 50 per 1000 live births [7] in India. Literacy rate is 79.3% [8].

## Methods

The conceptual framework for this work was based on the normalization process theory [9]. Hence focus of this work was on community care givers and government health care providers of CAP. The aim was to increase awareness in caregivers about CAP and built their trust in government health care providers. Augmentation of capacity and skills of government facilities and providers respectively, for treating CAP, was done. These would ensure that this intervention would be effective and was carefully monitored.

It was hypothesized that strengthening of existing public health system to provide sustainable quality care for cases of CAP followed by creating community awareness about CAP generally and demand for government services through strategic dissemination of the above mentioned validated messages to community may change care seeking behavior for CAP within 12 months. This could be measured by 50% improved utilization of services from qualified public health care providers. Hence an innovative package of “Community Orientation” of doctors and grass root health workers was developed where in they were informed about barriers to qualified care seeking that existed in the community and then showed them the validated messages developed earlier. In addition, their prior in service training on recognition and management of CAP was reinforced. Infrastructural strengthening of existing public health facilities was done.

Outcome measure was the number of CAP cases treated by Auxillary Nurse and Midwife (ANM)/doctors with medicines from pneumonia drug kits (PDK) or treated at Pneumonia Management Unit (PMU)/Pneumonia Management Corner (PMC).

### Study setting

The study was conducted in rural areas of Lucknow, which is capital of the state of Uttar Pradesh in Northern India. Rural Lucknow covers an area of approximately 2095.4 km^2^, has a population of 15,50,842 and is divided into 8 rural administrative units (blocks) [8].

Each rural block has at least one 30 bedded community health centre (CHC), which provides specialty out-patient and in-patient medical care, including pediatric care, to a population of 1,20,000 [10]. Under each CHC are 6 bedded Primary Health Centers (PHCs), also providing out-patient and in-patient care to a population of 30,000 [11]. In Lucknow, there were 9 CHCs and 33 PHCs during the study period.

ANM and Accredited Social Health Activist (ASHA) are the grass root level health workers, for a population of approximately 5000 (five villages) and 1000 (one village), respectively. In CHC and PHC, routine immunization (RI) clinic is run by ANM on every Wednesday and Saturday. Care givers bring their children here from neighboring villages. Once 5–10 children collect, ANM opens the vaccine and immunizes them. This rural health facility (CHC and PHC) based RI clinic provides a platform to the ANM to interact with the community and provide them behavior change intervention package. In villages, on village health and nutrition day (VHND), once a month, outreach immunization is done by ANM. ASHA mobilizes the community for this activity. Women aggregate in groups of 5 to 20. RI and VHND provide a platform to the community to interact freely with ANM and ASHA and obtain health related services and information [12].

### Study design

This was a community based open labeled behavioral trial conducted in 2 by 2 factorial design after obtaining prior institutional ethical clearance. The project was registered at “The American Economic Association’s Registry for Randomized Controlled Trials” (Registration number: AEARCTR-0003137) retrospectively. This was a pragmatic behavior trial, no interventional drug was used and all the cases of CAP received treatment as recommended by the WHO [3].

**Fig. 1:**
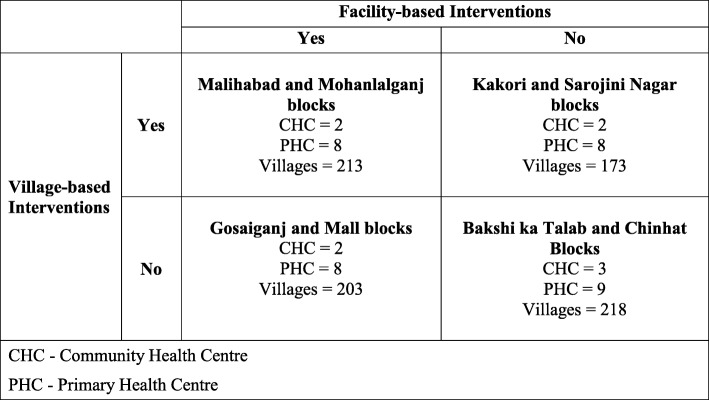
Design of Study

There are 8 rural administrative blocks in Lucknow district. Data on number of villages in each block was obtained from 2011 census [8], as this is roughly equal to the number of ASHA workers. Blocks with similar number of villages were purposively paired, like Mall (87 villages) with Gosaiganj (116 villages), Sarojini Nagar (90 villages) with Kakori (83 villages), Malihabad (100 villages) with Mohanlalganj (113 villages) and Chinhat (57 villages) with Bakshi ka Talab (161 villages). Then by random draw the pair of blocks was assigned to each intervention arm” (Fig. 1). There was one village-based and one facility-based intervention arm and each had corresponding comparator, which received usual care. Intervention was Pneumonia Awareness Sessions (PAS) [13]. In PAS, community awareness was created by using materials developed by same investigators. These were (a) text messages in form of three posters, (b) five real life stories of pneumonia with favorable and adverse outcomes as posters and story books and corresponding animated films, (c) four audio messages and (d) three video messages [4, 5]. The messages were targeted at early recognition of symptoms of pneumonia, recognition of danger signs and informing caregivers about risks of delayed treatment and promoting early and qualified care seeking from government/Public health facilities. At village level during VHND only text messages were used but at facility level during RI at CHCs/PHCs text as well as audio and video messages were used.

### Procedure

#### Interventions

PAS at facility-based interventions were conducted by ANM, on RI day, once in a month, at CHC/PHC. On the day of PAS, audio and video messages using speaker and projector were played / projected by research staff. Posters were hung on the walls. Parents of children attending RI were requested to stay till there was a small group of 10 to 20. They were first shown the video films and messages and then ANM explained about symptoms of pneumonia, importance of early care seeking and treatment from public health facility. This was reinforced while explaining the posters hung on the walls. Story books were also given to the participants to read or read out to them. In this manner one to three such sessions were conducted in a day. A list of participants attending the PAS was maintained by ANM. A usual session lasted for about 30 to 35 min.

PAS at village-based interventions were conducted by ASHA, on VHND once a month. Posters were hung on the walls at the place of VHND, which could be a school facility or her home. Group was formed by collecting 10–20 mothers attending VHND. ASHA explained the posters to the participants informing them about symptoms of pneumonia, importance of early care seeking and treatment from public health facility. Story books were given to the participants to read or read out to them. Two or three such sessions were conducted in a day. ASHA maintained a list of participants attending the session. A usual session lasted for about 15 to 20 min.

Monitoring adherence to PAS was done by: (a) Direct observation of PAS by research staff, primarily in facility-based intervention, (b) Interview a few participants of PAS. Their names were abstracted from the list maintained by ASHA in village-based intervention. For this research staff visited a particular village/ASHA once in 3 months and interviewed 2–3 participants, who had attended the last PAS. They enquired about its duration and number of posters displayed, story books given to read or read out and an informal recall of information given during PAS. Monthly telephone calls were also made to ASHAs by project team in the village-based intervention to get an update on scheduled PAS. In addition, villages were visited to confirm display of posters and story calendars at strategic locations in the village. In villages where posters and story calendars, were not displayed, ASHAs were requested to do so.

#### Infrastructural strengthening of existing public health facilities

This was done by establishing PMU at CHC and PMC at PHC. For this two existing beds were earmarked at CHC and one bed at PHC for managing cases of pneumonia by the state administration on request of investigators. Project provided pulse oximeter, baby mask with spacer and PDK since these were not available that time. The State Government was requested to ensure availability of (a) doctors and nurses round the clock (b) supply of injectable antibiotics such as ampicillin and gentamycin (c) oxygen necessary for management of CAP.

PDK were specially packaged by the project. PDK had amoxicillin (250 mg) dispersible tablets [3], paracetamol (500 mg) tablets and an instruction card describing their use in local language (Hindi) and having pictorial representation of symptoms of pneumonia. Two types of PDK were prepared. Green PDK was for children between 2 months to 12 months of age and had 10 dispersible tablets of amoxicillin (250 mg), 4 tablets of paracetamol (500 mg) and an instruction card. Yellow PDK was for children between 12 months to 59 months of age and had 20 dispersible tablets of amoxicillin (250 mg), 4 tablets of paracetamol (500 mg) and an instruction card. PDKs were supplied to CHCs, PHCs and ANMs through CHCs for distribution to cases of CAP. Feedback of 10% of mothers whose child was treated with PDK was collected about effectiveness and adverse effects of PDK from January 2017 to March 2018.

#### Trainings

Project team and government health workers were trained by project investigators. The investigators had already been trained earlier in Facility based Integrated Management of Neonatal and Childhood Illness(F-IMNCI) [14] run by Ministry of Health & Family Welfare, Government of India.

Project field team consists of one project coordinator, having more than 5 years of clinical research experience, one field investigator having experience of social work in rural areas, four field workers with social work background along with 1 year of experience in community health research project and a pharmacist. Investigators oriented the team about project and logistics, trained them using standard operating procedures (SOPs) for data collection (baseline/endline surveys, health facility audits), conduct and monitoring of PAS and for training of ASHAs.

The objective for trainings of Medical Officers, ANMs and ASHAs was to orient them about project, infrastructural strengthening activities undertaken, management of sick child (2 to 59 months of age) having cough with fast breathing and reporting of possibly CAP cases in a simple project format. Medical officers were given refresher training on Acute Respiratory Illness (ARI) module of F-IMNCI [14]. ANMs and ASHAs were trained on ARI module of IMNCI. IMNCI training videos were also used during the training. Role play exercises were carried out by ANMs & ASHAs for conducting PAS at Facility and Community level respectively.

At time of project initiation, 68 Medical Officers including Pediatricians (*n* = 6), medical doctors (*n* = 34) and doctors of indigenous system of Medicine (Ayurvedic, Yoga and Naturopathy, Unani, Siddha and Homeopathy) (*n* = 28) were posted at CHCs and PHCs in 8 rural blocks of Lucknow. In these blocks 344 ANMs and 1431 ASHAs were posted.

Trainings of Medical Officers, ANMs and ASHAs were conducted during pre intervention phase (October 2015 to July 2016). Project Investigators trained 59/68 (87%) Medical Officers in two separate batches at King George’s Medical University (KGMU). Trainings of ANMs were conducted in nine separate batches at their respective CHCs. Project Investigators trained 302/344 (88%) ANMs. Trained project team and residents from KGMU conducted training of 1236/1486 (83%) ASHAs in thirty four batches at their respective health facilities.

Refresher trainings were also conducted a year later. Refresher training for Medical Officers was conducted at KGMU, which was attended by 31/69 (45%) participants. Since some medical officers could not leave their facility to attend the training, one to one training was imparted to remaining medical officers at their work station by investigators. Refresher trainings for ANMs were organized at their respective CHCs in ten separate batches which were attended by 292/327 (89%). Retraining of 1203/1431 (84%) ASHAs were conducted at CHCs in thirty four batches.

#### Baseline and Endline surveys

Baseline survey was conducted to assess mothers’ knowledge about symptoms of CAP and care seeking for it. Socio-demographic characteristics of households were also captured. WHO’s thirty cluster sampling technique was used for selection of villages for base line survey. Census 2011 data was used to stratified villages as small (having up to 200 households), medium (having 201 to 400 households) and large villages (having 401 or more households). In each block, 10 villages from each stratum were randomly selected for survey.

In selected villages ASHAs were contacted and a list of households having child/children between 2 to 59 months of age was obtained. An approximate geographic mid point of the village was identified and then village was arbitrarily divided into east, west, north and south quadrants. Survey was conducted in two households, one farthest and other closest to the midpoint in each quadrant. The inclusion criteria were that the occupants were residing in the house for more than 6 months, would be staying in the house for next 1 year and having child/children between 2 to 59 months. A voluntarily written informed consent was obtained from head/acting head of household before survey.

Endline survey was conducted in same villages and in same households, in which baseline survey was done. In situations where a household now was not having any child between 2 to 59 months of age or the family had migrated or the household refused consent for participation, the adjacent household fulfilling all inclusion criteria was requested for participation. Data was collected on pre designed, validated questionnaire. Information was collected by face to face interview of mothers of eligible child. Variable for data collection were on socio-demographic status of household, information on possible pneumonia morbidity and knowledge of mothers about symptoms and danger signs of pneumonia.

#### Quality assurance

Robust mechanisms were employed to ensure quality of data collected during interviews and during adherence checks. For this, field visits were made by project coordinator to supervise and cross check the data collection during surveys and adherence checks. Investigators themselves validated 10% of data collected.

### Data management and statistical analysis

Data was collected by validated questionnaire. After data collection, it was entered in MS Excel and analyzed by using SPSS version 18 [15]. Exploratory data analysis was performed for outlier detection and missing observations. Descriptive statistics for continuous variables and frequency distribution for categorical variables were generated and compared for intervention wise variability from base line to end line. Univariate distribution was assessed for all study variables. Bivariate distribution was assessed for outcome variables by frequency counts and percentages. Chisquare test for proportion was used for testing the hypothesis. 95% confidence interval was calculated for the proportions. A *p* value of < 0.05 was taken as statistically significant using a two-tailed distribution.

## Results

The study was conducted from October 2015 to September 2018.

### Baseline survey

Baseline survey was conducted from February to May 2016. Total population of 13,515 was surveyed of which 49.8% (6729/13515) were males. Since there was no difference in socio-demographic variables by intervention, hence population characteristics are reported only. There were 57% (1369/2400) cemented, 18.5% (445/2400) mud and 24.5% (586/2400) households with mixture of both. Almost all households used multiple fuels for cooking. Biomass fuel like wood and dung cake was used by 79.5% (1907/2400) and 72.4% (1737/2400), households respectively. In addition, 40% (962/2400) households also used liquefied petroleum gas for cooking. Hand pumps were the main source of drinking water for 68.7% (1650/2400) households. Only 11.2% (270/2400) households had access to piped water supply. Separate kitchen was present in 47.2% (1133/2400) households, electricity was available in 77% (1848/2400) and toilet was present in 29.1% (698/2400) households. Main source of income was agriculture in 42.2% (1014/2400) households, business in 11.4% (273/2400) and professional activities in 8.2% (197/2400) while rest 38.2% (916/2400) were daily wages.

In 2400 households surveyed, there were 3351 eligible children. Table 1 gives base line characteristics for care seeking and maternal knowledge about CAP of study population by interventions.

**Table 1:** Baseline characteristics including care seeking and maternal knowledge about community acquired pneumonia of study population (*n* = 13,515) by intervention types

Characteristics of Study Population	Facility-based Intervention			Village-based intervention		
	Yes	No	*p* value	Yes	No	*p* value
Populations Surveyed n (%Male)	3356(49.8)	3373(50.1)	0.76	3365(50.0)	3364(49.9)	0.99
Eligible children 2–59 months age n(%)	1659(49.5)	1692(50.4)	0.42	1700(50.7)	1651(49.2)	0.23
Gender distribution of eligible children						
Children n (%Male)	877(52.8)	874(51.6)	0.92	875(51.4)	876(53.1)	0.07
Age distribution of eligible children						
2–11 months n (%)	425(25.6)	437(25.8)	0.89	447(26.2)	415(25.1)	0.44
12–23 months n (%)	425(25.6)	402(23.7)	0.21	422(24.8)	405(24.5)	0.84
24–59 months n(%)	809(48.7)	853(50.4)	0.34	831(48.8)	831(50.3)	0.40
Cases with possible pneumonia and their treatment providers (multiple responses)						
Children Suffered with possible pneumonia in last one year n (%)	392(23.6)	432(25.5)	0.20	420(24.7)	404(24.4)	0.87
By Home Remedies n (%)	117(29.8)	150(34.7)	0.14	121(28.8)	146(36.1)	0.03
By Medical store Keeper n (%)	16(4.0)	5(1.1)	0.01	18(4.2)	7(1.7)	0.03
By Government Physiciann (%)	21 (5.4)	12 (2.8)	0.06	14 (3.3)	24 (5.9)	0.05
By Private Physician n (%)	269 (68.6)	293 (67.8)	0.81	297 (70.7)	260 (64.4)	0.05
Knowledge of Mothers about symptoms of possible pneumonia						
Mothers Surveyed (N)	1231	1238	–	1239	1230	–
Mother who heard about pneumonia n /N (%)	1067/1231 (86.6)	1115/1238 (90.1)	0.01	1092/1239 (88.1)	1090/1230 (88.6)	0.71
Fast Breathing n (%)	150(14.1)	156(13.9)	0.96	158(14.5)	148(13.5)	0.55
Chest In drawing n (%)	164(15.3)	169(15.2)	0.89	147(13.5)	186(17.1)	0.02
Inability to drink n (%)	69(6.4)	59(5.3)	0.24	63(5.7)	65(5.9)	0.85
Lethargic n (%)	32(3.0)	18(1.6)	0.01	14(1.3)	27(2.4)	0.04

### Adherence checks

**Fig. 2:**
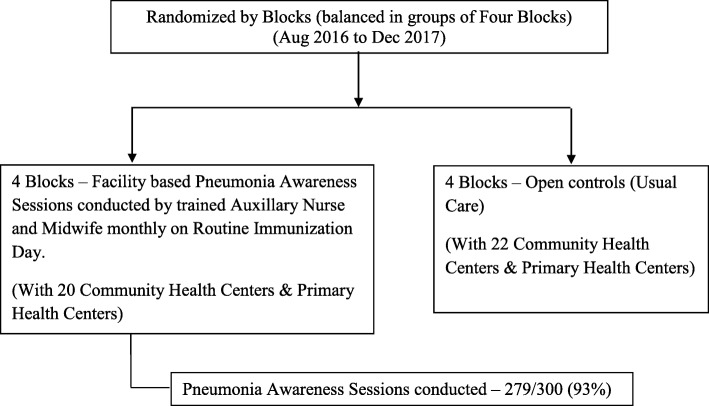
Flow diagram: Facility-based intervention

**Fig. 3:**
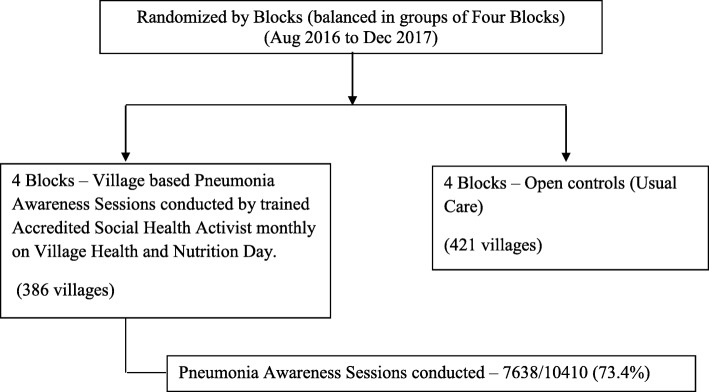
Flow diagram: Village-based intervention

PAS were conducted from August 2016 to December 2017. Adherence to PAS at facility-based intervention was 93.0% (279/300) as shown in consort diagram (Fig. 2) and to village-based intervention, it was 73.4% (7638/10410) as shown in consort diagram (Fig. 3).

Regular monitoring of PAS was done. Direct observation was done for 100% (279/279) of facility-based PAS and for 3.5% (267/7638) of village-based PAS. Monitoring through interview of participants, who had attended the PAS conducted by ASHA at village level, was done in all 696 villages.

Exit interviews of 5781 participants [28.4% (1644/5781) in facility-based and 71.6% (4137/5781) in village-based intervention] were conducted after PAS. Participants responded that they had gained information about symptoms of pneumonia like fast breathing [51.1% (2954/5781)] and chest in-drawing [50.63 (2927/5781)] during the sessions.

In an exit interview, a participant responded that “*During the session, ASHA told us about symptoms of pneumonia like cough with fast breathing and chest in drawing. If any of these symptoms is seen in our children, he/she must be immediately taken to a good hospital rather than going to traditional healers.*” Another participant said, “*Session was very useful as I gained information about pneumonia. I will go back to my village and spread awareness about pneumonia there.*”

From July 2016 to October 2017, 7730 cases of CAP were treated by PDK at PMU and PMC. In facility-based interventions 38.4% (3961/10306) and in village-based intervention 29.9% (3769/12604) cases were treated by PDK. Rest of the cases was treated by medicines supplied by government. Follow up of 717 children treated with PDK was done and their mothers were interviewed. It was found that 85% (609/717) children improved while 15% (108/717) took treatment from other providers. Adverse effects reported with PDK were diarrhea in 3.9% (28/717) and rashes in only 0.4% (3/717) children.

### Endline survey

Endline survey was conducted from December 2017 to April 2018. In endline survey 18% (432/2400) old households were replaced as they did not have any child between 2 and 59 months of age. Results of endline survey are given in Table 2.

**Table 2:** End line characteristics of study population including care seeking and maternal knowledge about community acquired pneumonia (*n* = 13,310) by intervention types

Characteristics of Study Population	Facility-based Intervention			Village-based Intervention		
	Yes	No	*p* value	Yes	No	*p* value
Populations Surveyed n (%Male)	3283(49.7)	3343(49.8)	0.30	3280(49.2)	3364(49.8)	0.15
Eligible children 2–59 months age n(%)	1677(49.1)	1694(49.6)	0.68	1735(50.8)	1677(49.2)	0.16
Gender distribution of eligible children						
Children n (%Male)	921(54.9)	883(5.1)	0.10	923(53.2)	881(52.5)	0.70
Age distribution of eligible children						
2–11 months n (%)	240(14.3)	215(12.6)	0.17	213(12.3)	242(14.4)	0.06
12–23 months n (%)	287(17.1)	287(16.9)	0.89	300(17.1)	274(16.3)	0.46
24–59 months n (%)	1161(69.2)	1192(70.4)	0.47	1222(70.4)	1161(69.2)	0.45
Cases with possible pneumonia and their treatment providers (multiple responses)						
Children Suffered with possible pneumonia in last one year n(%)	296(17.6)	316(18.6)	0.45	311(17.9)	301(17.9)	0.99
By Home Remedies n (%)	117(39.5)	150(47.5)	0.05	121(38.9)	146(48.5)	0.02
By Medical store keeper n (%)	11(3.7)	5(1.58)	0.10	12(3.8)	4(1.3)	0.05
By Government Physiciann (%)	53 (17.9)	33 (10.4)	0.005	50 (16.1)	36 (12.0)	0.14
By Private Physician n (%)	229 (77.4)	270 (85.4)	0.01	245 (78.8)	254 (84.4)	0.07
Knowledge of Mothers about symptoms of possible pneumonia						
Mothers Surveyed (N)	1228	1230	–	1237	1228	–
Mother who heard about pneumonia n /N (%)	1207/1228 (98.2)	1206/1230 (98.0)	0.66	1220/1237 (98.6)	1193/1228 (97.1)	0.01
Fast Breathing n (%)	493(40.8)	474(39.3)	0.44	664(54.4)	303(25.3)	< 0.0001
Chest In drawing n (%)	530(43.9)	456(37.1)	0.002	642(52.6)	344(28.8)	< 0.0001
Inability to drink n (%)	313(25.9)	358(29.6)	0.04	450(36.8)	221(18.7)	< 0.0001
Lethargic n (%)	62(5.1)	64(5.3)	0.85	88(7.2)	38(3.1)	< 0.0001

In 2400 households surveyed, 3412 children were found to be eligible with equal distribution of age categories and gender across all interventions as was seen in baseline survey. At endline survey, 2353/3412 (69.9%) as compared to 1662/3351 (49.6%) [*p* < 0.0001] children at base line were between 24 and 59 months of age. We interviewed 2465 mothers at endline and found that there were a significant increase in knowledge of mothers about symptoms and danger signs of pneumonia, across all interventions (*p* < 0.0001).

Table 3 shows changes in utilization of government health facilities for the treatment of possible pneumonia. In village-based intervention an increase from 3.33% at baseline to 16.1% at endline was observed. Facility-based intervention had an increase from 5.4 to 17.9% from baseline to endline. Village-based intervention has statistically significant increase in utilization of government health facilities as compared to facility-based intervention (*p* < 0.0001). The change was similar in both gender and was not affected by age of mother.

**Table 3:** Change in health seeking behavior for childhood pneumonia at government health facilities from baseline to endline by interventions

Treatment for possible pneumonia at government health facilities	Facility-based Intervention		Village-based Intervention	
	Yes	No	Yes	No
Baseline n/N (%)	21/392 (5.35)	12/432 (2.8)	14/420 (3.33)	24/404 (5.9)
Endline n/N (%)	53/296 (17.9)	33/316 (10.4)	50/311 (16.1)	36/301 (12.0)
% change in the treatment for possible pneumonia at government health facilities (95% CI)	68.9% (64.4–73.6)	73.5% (68.8–77.2)	79.3% (75.1–82.9)	50.3% (45.1–54.9)
*p* value	0.02		< 0.0001	

n = Total children taken treatment for possible pneumonia from government health facilities

N = Children Suffered with possible pneumonia

## Discussion

This open labeled behavioral trial was conducted in rural Lucknow India, over a period of 36 months (from October 2015 to September 2018). In the current study it was observed that healthcare seeking from government health facilities for CAP increased significantly from baseline level of 3.33% to endline level of 16.1% (improvement = 79.3%; p < 0.0001), when community was targeted by village-based intervention. At baseline, private health care providers were preferred and were approached by more than two-third of parents for treatment of CAP, in comparison to government health care providers, who were approached by less than one in twenty. By the end of the study, in addition to visiting private health care providers for CAP, the community now increasingly also visited government facilities in both the arms and the change was statistically significantly greater in the village-based intervention arm. A period of 1 year was too short to see massive shifts in provider preferences, yet it was possible with repeated reinforcements in the village-based arm. Concomitantly, significant change was also observed in the knowledge of mothers about symptoms of pneumonia such as, fast breathing and chest in drawing (Tables 1 and 2) in village-based intervention arm which was not seen in facility-based intervention arm.

Robust methods were used for the study. Thirty cluster sampling technique recommended by WHO was used to select villages for baseline and endline surveys. Ten percent data collection process was monitored for quality assurance. Adherence to PAS was good in both interventions. Regular monitoring of PAS sessions was done to ensure quality.

This work was based on normalization process theory [9]. Hence it proceeded from the care providers and care givers coherently recognizing the problems in managing CAP in rural communities. This was facilitated by sharing real life stories of cases of CAP who had adverse outcomes. Thereafter, through behavioral intervention, IEC material promoted cognitive participation in CAP detection and treatment. Infrastructural strengthening of health facilities and PAS promoted collective action against CAP. Lastly endline survey was done and results were shared with stakeholders. This reinforced good practices and their integration into the system. It also lead to change in health care seeking practices for CAP by community in village-based intervention arm. For health care providers, this study leads to integration of quality care for CAP which resulted in their earning public trust.

In India, healthcare is provided in government or public sector as well as in private sector. While government sector physicians have to be qualified in modern medicine to prescribe drugs, private sector has both qualified physicians and unqualified health care providers. It is almost impossible to ascertain who is a qualified physician in private setting [4]. Hence it was assumed that qualified physicians were only in government sector, acknowledging that this would be an underestimation.

Behavior change and educational interventions have resulted in improvement in healthcare setting practices. In a review article, Darmstadt et al. concluded that health education of families and communities create demand for skilled care [16]. Likewise Kumar et al. conducted a community based behavior change study to assess impact on neonatal mortality in an area close to Lucknow district, in the same state Uttar Pradesh. Socio-economic status and cultural beliefs were similar to those in the current study area. They interpreted that a socio-culturally contextualized, community based intervention, targeted at high-risk newborn care practices, can lead to substantial behavioral modification and reduction in neonatal mortality [17].

In a systemic review of randomized controlled trials, Das et al. reported that community based interventions have the potential to scale up health care seeking and the use of essential commodities and significantly decrease morbidity and mortality burden due to diarrhea and pneumonia in children under the age of 5 years [18]. Similar observations were seen in current study. Shaikh et al. demonstrated in their study in Jamshoro, Pakistan, that educating mothers had resulted in improved immunization of children, improved continued breast feeding during illness and increased ability of mothers to recognize danger signs. They further emphasized that effects of such interventions do not last for long periods and require continued efforts if changes in attitude and behavior are to be sustained [19]. These studies have found results similar to that reported in the current study.

Awasthi et al. observed that one to one counseling of mothers at facility level in the immediate postnatal period led to significant improvement in qualified medical care seeking for sick newborns [20]. Unlike their study, the current study reflects that facility-based intervention alone was neither able to bring statistically significant change in healthcare seeking behavior for CAP nor resulted in any statistically significant change in knowledge of mothers about symptoms and danger signs of pneumonia. This may be because PAS in facility-based intervention were given to families residing in villages near the facility only.

### Strengths

This community based behavioral trial was conducted in partnership with the government and under pragmatic conditions. There was good adherence to intervention, which was PAS. PDK was a new concept for the public health system as well as for the community. Presentable packing, full course of medicines and instruction card with information on usage and follow up of child were few features of PDK that helped in building trust of community on public health system.

### Limitations

This was a two by two factorial design cluster randomized trial, but the number of clusters was small in each arm. Due to close proximity of study blocks, there was a possibility of contamination of interventions across each other. Although, if this had happened, it would have resulted in spread of awareness about CAP, which was beneficial to community. But it potentially biased the results towards null. Baseline and endline survey were conducted in same set of villages across all arms. Since the community was not aware about the objectives of the study their responses about choice of healthcare provider were likely to be unbiased.

### Challenges

The significant challenge during study was to maintain adherence to PAS at village level. This was ensured by collaborating with the official managers of ASHAs at block level and with Medical Officers who ensured timely conduct of PAS. ASHAs who were conducting PAS regularly were awarded certificate of appreciation. Another challenge was attrition of trained Medical Officers due to transfers within the state. To counter it, one to one training was provided to newly joined Medical Officers at their health facilities. An important challenge was that infra-structurally facilities at CHC were not adequately equipt to manage cases of CAP. Therefore infrastructural strengthening was done by providing pulse oximeter, baby mask with spacer, salbutamol inhaler and PDK before giving interventions. Further, facilities had practice of maintaining medical records manually and in large proportion of cases diagnosis was not written or was illegible. While this challenge was identified it could not be rectified during the course of the project. Hence the actual number of cases of CAP attending the facility could not be reported.

## Conclusion

Conduct of structured PAS using validated IEC material at village level with infrastructural strengthening resulted in improved qualified care seeking from government facilities for CAP.

### Recommendation

This study established a proof of principle that community awareness creation would bring about a change in healthcare seeking behavior and further large scale studies can be planned. The sustainability of PAS depends upon the motivation and commitment to fight CAP among health workers. Since project had created demand and improved the supply of quality of care for CAP, this can be scaled up from here by the government. The IEC materials are shared with government and are also available at website (www.fightpneumonia.org) as open access material.

## Data Availability

The datasets used and/or analyzed during the current study are available from the corresponding author on reasonable request.

## Abbreviations

ANM: Auxillary Nurse and Midwife
ARI: Acute Respiratory Illness
ASHA: Accredited Social Health Activist
BPM: Breaths per Minute
CAP: Community Acquired Pneumonia
CHC: Community Health Centre
FIMNCI: Facility based Integrated Management of Neonatal & Childhood Illness
IEC: Information education and communication
KGMU: King George’s Medical University
PAS: Pneumonia Awareness Session
PDK: Pneumonia Drug Kit
PHC: Primary Health Centre
PMC: Pneumonia Management Corner
PMU: Pneumonia Management Unit
RI: Routine Immunization
SOP: Standard Operating Procedure
VHND: Village Health and Nutrition Day
WHO: World Health Organization
